# Hospital Statistics. II.—A Suggested Remedy for the Present Chaos

**Published:** 1913-11-01

**Authors:** 


### HOSPITAL STATISTICS.

### II.—A Suggested Remedy for the Present Chaos.

The officers entrusted with the supervision of statistical records in the teaching hospitals are generally termed registrars. There are usually at least two, and often three or more, recruited from among those younger graduates who, having attained special academic distinction, hope ultimately to obtain posts on the visiting staff. Although nominally statistical officers the registrars are in fact clinicians, supervise the work of the clinical clerks and dressers, and act as *locum tenentes* for the visiting staff in the out-patient clinics. The registrars are naturally brought into intimate contact with the statistics of the hospital, but the only purely statistical work, apart from extending and correcting the raw material by themselves, supplementing the notes of house physicians and clerks, which regularly falls to their lot, is of the following kind. If a member of the visiting staff conceives that a certain treatment adopted in his wards influences the course of any disease, he will probably request the registrar to furnish him with a statistical return, comparing the results of treatment in his own wards with the ordinary experience of the hospital, the object being to determine whether the treatment has had beneficial results. It will be as well to consider just what the satisfactory performance of this task involves.

We may suppose the problem to be merely whether there be a difference between the rates of case mortality (*i.e.*, the fatality) exhibited in the treated and untreated series. *If the questions of age, sex, and occupation do not arise*, then the probability that the difference, if any, is not a chance event unconnected with the treatment can be calculated. The method of computation depends, *inter alia*, upon the respective magnitudes of the two series and upon the rates of fatality. A process which is applicable when the rates are high, say not less than about 10 per cent., cannot be used without serious risk of error when the fatality, although very different in the two cases, is absolutely small. The choice of a suitable process, even in so artificially simplified a case, involves some measure of experience and technical knowledge; the mechanical application of a formula cannot produce satisfactory results. But the simplification postulated really takes the case outside the region of practice altogether; in real life it is hardly ever possible to neglect age, occupation, and other factors. An evaluation of the effects exerted by such factors may task the ingenuity of a highly trained statistician, involving as it does considerable technical skill. What is the probability that the registrar will possess this skill? It is very small indeed; in fact, he is unlikely to be able to deal with even the simpler problem described. It might in truth be argued that had he possessed such skill he would not have been registrar at all. The mental type which excels in clinical work is different from that which delights in statistical calculations; even were this not so, it would be asking too much of human nature to expect that a young man desirous of becoming a medical or surgical registrar would postpone his candidature until he has attended a course of instruction in statistical methods, especially since he has no reason to suppose that the acquisition of statistical knowledge will help him to obtain either the post he is immediately seeking or that on the visiting staff which is the ultimate goal of his ambition. As a matter of fact the only stage of the investigation that the present-day registrar is competent to undertake is the extraction of the figures, a process which he can, and frequently does, leave to a clerk. If we agree to call the registrar the medical statistician of a hospital, we see that the organisation is similar to that of the scientific departments of the medical schools in the bad old days when a man was appointed to teach physiology, not because he was a promising physiologist or a good teacher, but to enable him to retain some sort of connection with the hospital pending a vacancy on the clinical staff. The position is really less favourable to statistics than was the older arrangement to physiology, since a moderately conscientious man appointed to *teach* a subject will certainly endeavour to acquire some knowledge of it. A conscientious registrar will feel that his clinical duties are of much more importance than his statistical tasks and will have little or no inducement to learn the elements of statistical science.

It may seem that the remedy is to appoint a trained statistician to deal with the hospital statistics, just as a trained bacteriologist is appointed to carry out the bacteriological investigations required. Undoubtedly such a change would speedily lead to the removal of the weaknesses in the present system which have been pointed out; it is, however, by no means free from objection. The number of adequately remunerated statistical posts in existence, even including those in the public offices, is very small; consequently, the value of a hospital post as a stepping-stone to something else would be slight, and it would be necessary to provide a considerably larger salary than falls to the lot of a registrar. Again, a statistician requires laboratory accommodation and assistance, involving further expenditure. It seems very doubtful whether the finances of the general hospitals are in a condition to bear the strain imposed by the establishment and staffing of medico-statistical laboratories, and the desired end could be as well or better attained by centralisation. Obviously a clinical bacteriologist must be appointed for each hospital; the nature of the work renders this imperative. But the remark does not apply with equal force to the statistician, since his material can be duplicated, while that of the bacteriologist cannot. A card-index system arranged on carefully thought out lines might allow of the main records being multiplied, and the extra clinical labour involved would not be very great. In this way the combined data of all the hospitals might be systematically arranged and analysed in a central bureau. The amount of material relatively to that dealt with in the statistical department of any great city is small, and its centralisation would be an enormous advantage to all research workers, not to speak of the resulting economy in staff and laboratory outgoings. This central bureau should be under the supervision of a statistical committee nominated by the authorities of the different hospitals concerned; each authority might be represented by one member of the medical staff and one member of the house committee. The permanent staff should consist of two or three trained medical statisticians, with a competent number of clerical assistants. The bureau should be entrusted with the publication of an annual report on the hospitals' experience, should deal with the statistical problems now either relegated to the registrars or not considered at all, should initiate research work, and, lastly, should both provide instruction in method and generally assist private workers.

The initial difficulties which confront any such scheme as the above are twofold. In the first place there is the question of expense, and in the second the difficulty of obtaining co-operation between different hospitals, the systems of which may not be uniform. With respect to the first point, we feel sure that were the charitable public acquainted with the facts as set forth—viz., that at present, so far as the experience of the great general hospitals is concerned, scientific statistics do not exist, and that annually much time, labour, and money are wasted in the compilation of data which, if not worthless, are at least so presented that their significance is obscure—we have little doubt that any practical scheme of reform would not be rendered abortive by lack of funds.

The second difficulty is more formidable. It is a fact that many members of hospital staffs regard hospital statistics as of no scientific value at all. Whether the exponents of such a view are really competent to form a judgment as to statistical matters is open to doubt. It may at least be remarked that if hospital statistics are not worthy of scientific treatment it is singular that so many eminent clinicians use them in lectures and memoirs to point morals or adorn tales. Perhaps the data when treated in accordance with the best statistical practice will not yield satisfactory conclusions; they certainly can but lead to error when dealt with as at present upon no recognisable principle at all. We are far from thinking that hospital data are free from errors or that a reform in the methods of their compilation and analysis would immediately put us in possession of great and unsuspected truths. We do, however, firmly maintain that the present state of chaos is incapable of defence. No hospital which handled its financial records as all hospitals treat their medico-statistical data could exist for twelve months. Many problems of importance can only be solved with the assistance of these neglected records. Lack of accommodation and other circumstances prevent the inmates of a hospital from being a random sample of the sick persons to be found in London. On the other hand, there are some important diseases which, either on account of their gravity or because institutional treatment is required, are in a large number of those cases which occur among the working-class population treated in hospitals.* The surgical treatment of appendicitis, for instance, is, so far as urban populations are concerned, mainly carried out in institutions.

An adequate statistical analysis of the immense numbers of cases of this kind which have been, and are being, admitted to the hospitals has never been undertaken. Again, we know from the decennial supplements of the Registrar-General that the mortality due to various named causes is strikingly different in different occupational groups. This must partly be due to an unequal incidence of the diseases, but it must also be affected by variations in fatality. Hospital statistics cannot give us full knowledge as to the fatality of all diseases among the working-class population, but would certainly throw light upon some of them. For this purpose a great collection of statistics is necessary, such a collection as would result from the pooling of all the hospital records. Of the influence of an hereditary factor in the origin of disease and upon the prognosis when the disease has arisen we say nothing. This is a subject of great importance and must necessarily be dealt with on statistical lines; but it seems to us doubtful whether hospital data, even if collected and analysed scientifically, would prove of service in this field.

It is rather in connection with occupational morbidity and the influence exerted on the course of disease by various therapeutic and dietetic measures that the analysis of hospital statistics seems to promise results of the highest value. It is not possible here to complete an examination of this important subject. There may be insuperable objections to any reform of hospital statistics. It is possible that these records are of value when cited piecemeal and without analysis by individuals, and would be worthless if collected on a large scale and subjected to the rigorous examination rendered possible by modern developments of method. If reasons for such a belief can be advanced it is high time they should be laid before the public. Nobody can doubt that at present either far too much or far too little is made of hospital results. Too much if the data are as bad as some hold; too little if they have a value at all commensurate with the energy displayed in collecting the raw material.

* Of 387 deaths from appendicitis registered in London in 1911, 268 (69.3 per cent.) occurred in hospitals or nursing homes; of 293 deaths from acute endocarditis 124 (42.3 per cent.) took place in hospitals or nursing homes. [See the Seventy-fourth Annual Report of the Registrar-General.]

